# Influence of the measurement method of features in ultrasound images of the thyroid in the diagnosis of Hashimoto’s disease

**DOI:** 10.1186/1475-925X-11-91

**Published:** 2012-11-28

**Authors:** Robert Koprowski, Anna Korzyńska, Zygmunt Wróbel, Witold Zieleźnik, Agnieszka Witkowska, Justyna Małyszek, Waldemar Wójcik

**Affiliations:** 1 Department of Computer Biomedical Systems, University of Silesia, Institute of Computer Science, Będzińska 39 str, 41-200, Sosnowiec, Poland; 2 Nałęcz Institute of Biocybernetics and Biomedical Engineering, Polish Academy of Sciences, Ks. Trojdena 4 str, 02-109, Warszawa, Poland; 3 Internal Medicine Practice, Dworcowa 25/5 str, 41-902, Bytom, Poland; 4 Faculty of Electrical Engineering and Computer Science, Lublin University of Technology, Nadbystrzycka 38 D str, 20 – 618, Lublin, Poland

**Keywords:** Image processing, Hashimoto, Thyroid, Ultrasonograms

## Abstract

**Introduction:**

This paper shows the influence of a measurement method of features in the diagnosis of Hashimoto’s disease. Sensitivity of the algorithm to changes in the parameters of the ROI, namely shift, resizing and rotation, has been presented. The obtained results were also compared to the methods known from the literature in which decision trees or average gray level thresholding are used.

**Material:**

In the study, 288 images obtained from patients with Hashimoto’s disease and 236 images from healthy subjects have been analyzed. For each person, an ultrasound examination of the left and right thyroid lobe in transverse and longitudinal sections has been performed.

**Method:**

With the use of the developed algorithm, a discriminant analysis has been conducted for the following five options: linear, diaglinear, quadratic, diagquadratic and mahalanobis. The left and right thyroid lobes have been analyzed both together and separately in transverse and longitudinal sections. In addition, the algorithm enabled to analyze specificity and sensitivity as well as the impact of sensitivity of ROI shift, repositioning and rotation on the measured features.

**Results and summary:**

The analysis has shown that the highest accuracy was obtained for the longitudinal section (LD) with the method of linear, yielding sensitivity = 76%, specificity = 95% and accuracy *ACC* = 84%. The conducted sensitivity assessment confirms that changes in the position and size of the ROI have little effect on sensitivity and specificity. The analysis of all cases, that is, images of the left and right thyroid lobes in transverse and longitudinal sections, has shown specificity ranging from 60% to 95% and sensitivity from 62% to 89%. Additionally, it was shown that the value of *ACC* for the method using decision trees as a classifier is equal to 84% for the analyzed data. Thresholding of average brightness of the ROI gave *ACC* equal to 76%.

## Introduction

The measurement of thyroid echogenicity is currently one of the most common and standardly performed measurements in ultrasound diagnosis. Measurements of this type have evolved over the years in accordance with progress and increase in the quality of ultrasound equipment. In the beginning [1-4], qualitative evaluation methods related to the areas of analysis and methods of description were explained. At that time, it was proved that normal thyroid echogenicity is higher than that of sternocleidomastoid and subhyoid muscles. Later, this approach was extended and the salivary gland was included in the analysis [5]. With advances in computer technology and capabilities of digital recording and analysis, first papers on quantitative measurements [6-9] appeared. Those measurements were related to the use of basic methods of image analysis and processing in the diagnosis of, for example, Hashimoto’s disease [10,11]. Due to imperfections introduced by the measurement method (scanning ultrasound pictures), this methodology has not been adopted in clinical practice. Scanning as well as other processes of non-digital image analysis introduce a significant error of the method and are not repeatable. The next stage were the methods of digital images analysis which ensured repeatability of measurement. They are mainly presented in Mailloux’s papers from the years 1984 to 1986 [12-14]. Those papers concern the application of texture analysis in ultrasound images. Nowadays, there are modern methods of analysis of ultrasound images. Although they are virtually limitless, there is still no clear method of disk image analysis that would give reproducible and unambiguous results. Many authors now attempt to use morphological and statistical methods in the analysis of texture of the thyroid lobe. In those methods, both the analysis of histograms, which gives partially correct results, as well as more advanced methods of texture analysis are used. These are, for example, methods [15,16] which are based on the analysis of the areas indicated by the operator. The areas are analyzed by Co-occurrence Matrices. Then, Haralick’s coefficients are determined. The analysis of the Radon Domain [17] or Fuzzifying the Local Binary Patterns [18,19] are further examples of the afore-mentioned methods. In recent works, an approach based on Support Vector Machines [20-22] can also be found. The results obtained using the Bayes classifier [23] or Gaussian mixture model [24] are interesting as well. In the literature, there are also other approaches to texture analysis, such as neural networks [25,26] other [27-33] or dissertation [34]. The methods of image analysis presented in those works need to be profiled to a specific application every time they are used. However, valuable evidence related to the measurement method and rough interpretation of ultrasound images of the thyroid arise from those works. For example, it was found that it is best to set the instrument to 10 MHz to achieve accuracy of results; the cut-off point is -69dB for Hashimoto’s disease [35,36]. The authors of papers [37,38] showed advanced methods of texture analysis of thyroid lobe images. Those methods were shaped to the diagnosis of Hashimoto’s disease. In paper [39], it was proved that only three of the ten features measured in an image are enough for a correct assessment of Hashimoto’s disease. These three features will be the basis of analysis in this paper.

## Material

In this paper, the examined group were:

  ― 59 healthy subjects aged 18 to 60,

  ― 73 patients with Hashimoto’s disease

The images were obtained with GE Logiq P5 ultrasound machine. The frequency of the transmitter was set to 10 MHz, and harmonic imaging option was turned off. All the images were recorded in DICOM format. During the test, the patient remained in the supine position and the doctor applied ultrasound heads to the right and left side of the thyroid.

For each subject, four ultrasound images were taken. Those were images of the right and left lobe of the thyroid in both transverse and longitudinal section. Due to thick errors caused by improperly performed acquisition, 288 images from patients with Hashimoto’s disease and 236 images from healthy subjects were further analyzed. The examined group was divided in equal proportions into learning, validation and test groups. Each ultrasound image was analyzed in great detail and, then, an expert physician selected for analysis a rectangular region (ROI) which covered the thyroid lobe in individual sections. Each time, the ROI included the greatest possible and most representative area of the patient’s thyroid lobe.

## Method

### Preliminary image analysis

*L*_*GRAY*_ input images were obtained from GE ultrasound machine with a resolution of *M*_*G*_×*N*_*G*_=614×816 pixels. The first stage of image preprocessing was filtration done with the use of a median filter whose mask size is *M*_*h*_×*N*_*h*_=3×3. The filtered images *L*_*MED*_ were further used in subsequent stages of image analysis and processing. In the images (taken in transverse and longitudinal sections of the right and left thyroid lobe), an expert physician selected a rectangular area of analysis. Papers [37] and [38] describe an automated way of selecting this area of the thyroid, but only in transverse sections. The basis for its operation is a clearly visible artery calibrating the recognition system. The manually marked area of the thyroid lobe *L*_*S*_ with a resolution of *M*_*s*_×*N*_*s*_ was analyzed. The results of the analysis are shown below.

### The measured image features

The analysis of the thyroid lobe as texture in paper [39] proved that only 3 out of 10 different features are reliable in the assessment of Hashimoto’s disease. These features are: smoothness- *w*(1), minimum brightness after removing noise- *w*(2) and the percentage number of areas 8×8 in the square-tree decomposition- *w*(3). The ways to calculate individual values of the features are discussed in detail below:

**w(1) – smoothness**

(1) $$\;w\left(3\right)=1-\frac{1}{1+{w_{\mathrm{STD}}}^{2}}$$

where w_STD_

(2) $$\;w_{\mathrm{STD}}=\;\sqrt{\frac{1}{M*N}\overset{m=1}{\underset{M}{\sum }}\sum_{N}^{n=1}(Ls\left(m,n\right)-\overset{―}{Ls}})$$

Smoothness defined by the formula (1) is relatively easy to interpret because it is a standardized measure based on a standard deviation of the mean.

**w(2) – the minimum value of brightness in the image Ls after removing all the pixels whose number for a given brightness is less than 20% of the largest number of brightness pixels, i.e.:**

(3) $$\;hist\left(i\right)=\sum_{m=1}^{M}\sum_{n=1}^{N}k\left(i,m,n\right)$$

where

(4) $$\;k\left(i,m,n\right)=\{\begin{array}{l} 1\;if\;Ls\left(m,n\right)=1\\ 0\;other \end{array}$$

for *i*=1,2,3,…,254,255.

(5) $$\;hist_{m}=\underset{i}{max}\;hist\;\left(i\right)$$

where

(6) $$\;hist_{w}\left(i\right)=\{\begin{array}{l} hist\left(i\right)\;if\;hist\left(i\right)>0.2*hist_{m}\\ hist_{m}\;other \end{array}$$

(7) $$\;hist_{w}\left(i^{*}\right)=\underset{i}{min}\;hist_{w}\left(i\right)$$

On the basis of pre-tests and preliminary analyses, a noise threshold of 0.2 was set. The value of i* formulated in this way constitutes another feature, i.e. *w*(2).

w(3) – percentage of instances of areas 8×8 obtained for the 10% threshold as a result of a square-tree decomposition.

**Figure 1:**
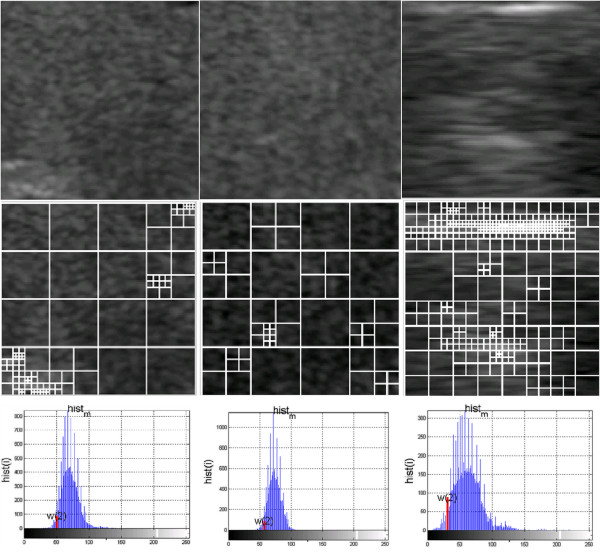
**Examples of textures of the thyroid lobes with images showing the square-tree division and a histogram with the value of the feature *w*(2).** For the presented images, there are values of the features *w*(1), *w*(2), *w*(3) - for the first column *w*(1)=0.0025, *w*(2)=51, *w*(3)=26.17, for the second column *w*(1)=0.0013, *w*(2)=58, *w*(3)=33.98 and for the third column *w*(1)=0.0081, *w*(2)=33, *w*(3)=15.67.

A square-tree decomposition [39,40] enables to determine some statistical characteristics of the image. In this case, these are areas of 8×8 resulting from a division of the image *Ls*. Their number is a measure of the feature *w*(3). The thyroid image *L*_*S*_ with a resolution of *M*_*s*_×*N*_*s*_ is divided into “*i*” rectangular areas *L*_*i*_ with a resolution of *M*_*i*_×*N*_*i*_ for the "*i*" coefficient value in the range 1<=*i*<=*I*. These areas can also have different sizes, i.e. 1×1, and the largest - *M*_*s*_×*N*_*s*_ pixels. However, for the adopted definition of the feature w(3) and the analyses carried out in [21], only the areas of 8×8 pixels are relevant. The 10% brightness threshold, which is the criterion of division into other smaller areas, was chosen on the basis of preliminary measurements and analyses of *Ls* image content [21]. An example of a division is shown in Figure 1. The values of the feature *w*(3) are calculated as a percentage relative to the whole image.

The features *w*(1) to *w*(3) are the basis for further analysis.

## Results

A qualitative assessment of the measurement of echogenicity and its impact on the results obtained in the classification of Hashimoto’s disease was conducted using a statistical approach [41,42]. A discriminant analysis was used for the following five options:

linear- linear discriminant analysis,

diaglinear- linear discriminant analysis but with a diagonal covariance matrix estimate (naive Bayes classifiers),

quadratic- quadratic discriminant analysis,

diagquadratic- quadratic discriminant analysis but with a diagonal covariance matrix estimate (naive Bayes classifiers),

mahalanobis- using the distance Mahalanobis with stratified covariance estimates.

It was assumed that the discriminatory variables *w*(1), *w*(2), *w*(3) represent a three-dimensional normal distribution (although previous studies carried out with the use of multivariate discriminant functions confirm the correctness of the classification, even in violation of this assumption). Divisibility of the variables is retained. This divisibility is reflected in the systematic difference in mean values between groups. Also the equality of covariance matrices is preserved. Empirical studies show that the assumption of equal group covariance matrices can be omitted.

These specific types of discriminant analysis were used to classify patients from healthy subjects. Assuming the classification results in terms of the following results: *TP*- true positive, *TN*- true negative, *FP*- false positive, *FN*- false negative, sensitivity was defined as *TPR* = *TP* / (*TP* + *FN*) and specificity as *SPC* = *TN* / (*FP* + *TN*). In addition, the analysis was performed for the following groups of data:

LO– images of the left transverse section of the thyroid,

RO– images of the right transverse section of the thyroid,

LRO– images of the left and right transverse sections of the thyroid,

RD– images of the right longitudinal section of the thyroid,

LD– images of the left longitudinal section of the thyroid,

RLD– images of the right and left longitudinal section of the thyroid,

RLOD– images of the right and left transverse and longitudinal sections of the thyroid.

**Figure 2:**
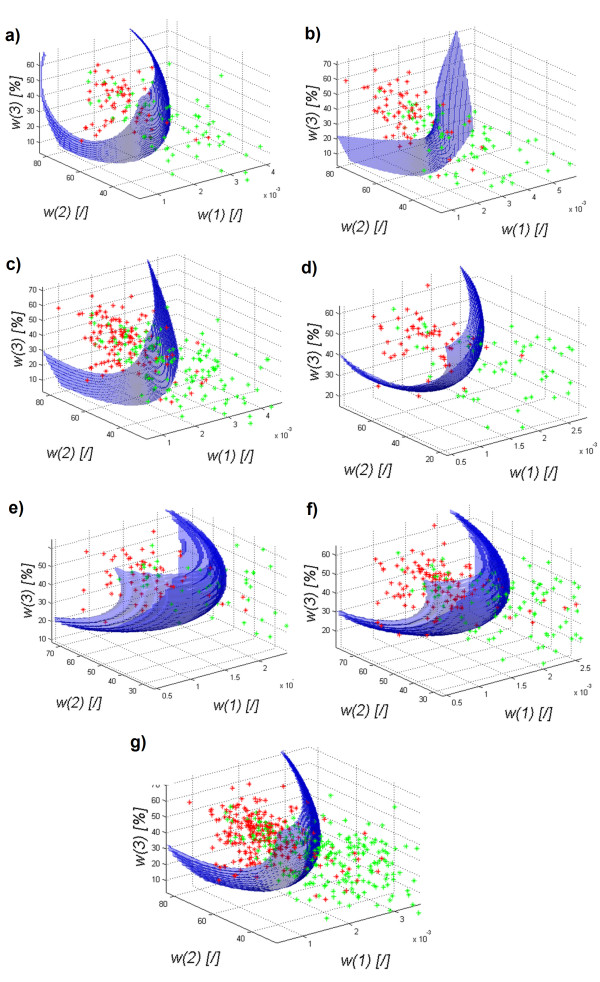
**The graph of the decision function for classification (quadratic) of healthy subjects (red) from patients (green) depending on the features *w* (1), *w* (2) and *w*(3) for the images of the: a) left transverse section of the thyroid LO, b) right transverse section of the thyroid RO, c) left and right transverse sections of the thyroid LRO, d) left longitudinal section of the thyroid LD, e) right longitudinal section of the thyroid RD, f) left and right longitudinal sections of the thyroid RLD, g) left and right transverse and longitudinal sections of the thyroid LROD.** The decision function was selected automatically and the classification gave the following results: graph **a)** specificity at 0.9 and sensitivity at 0.62, graph **b)** specificity at 0.87 and sensitivity at 0.74, graph **c)** specificity at 0.89 and sensitivity at 0.64, graph **d)** specificity was at 0.89 and sensitivity at 0.77, graph **e)** specificity was at 0.92 and sensitivity at 0.62, graph **f)** specificity of 0.89 and sensitivity of 0.7 and graph **g)** specificity is at 0.89 and sensitivity at 0.65.

The results obtained for the discriminant analysis - quadratic discriminant analysis – are shown in Figure 2a) to g).

**Figure 3:**
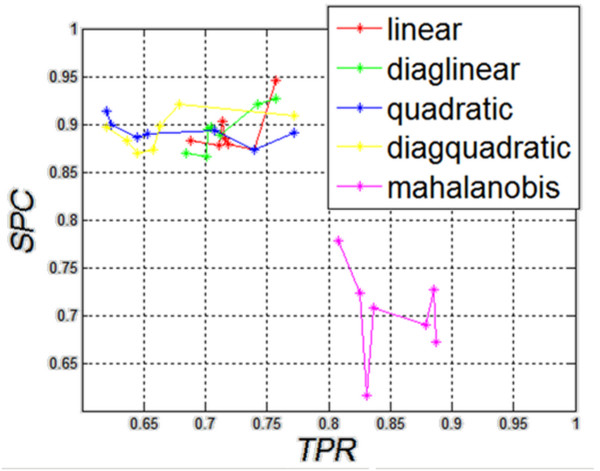
**The graph of specificity ( *SPC* ) as a function of sensitivity ( *TPR* ) for different types of classification.** As the graph shows, the results obtained for the linear diaglinear, quadratic and diagquadratic classifications are similar. Differences in sensitivity and specificity are clearly visible for the mahalanobis type. As shown later on, the calculated value of accuracy does not indicate clearly this type of classification as the best one.

The results in Figure 2a) to g) show that regardless of the origin of the analyzed images of the thyroid (left, right lobe), and regardless of the section (transverse, longitudinal), the shape of the decision function formed between classes is not basic. The results of specificity (*SPC*) and sensitivity (*TPR*) for different types of classification are presented in Figure 3, which shows that Mahalanobis type slightly stands out. For all cases, the shapes of a decision function for classification are similar. It means that combining various sections, for example, LO with RO, LD with RD, etc., is justified and may increase the value of *SPC* and *TPR*. The values for the best classifier can be seen in Table 1 - specificity and Table 2- sensitivity.

**Table 1:** Table showing the dependence of the results of specificity from the types of classification and the analyzed areas

	**LO**	**RO**	**LRO**	**LD**	**RD**	**LRD**	**LROD**
linear	0.883	0.873	0.878	0.945	0.879	0.902	0.885
diaglinear	0.866	0.888	0.869	0.927	0.896	0.920	0.894
quadric	0.900	0.873	0.886	0.890	0.913	0.893	0.889
diagquadric	0.883	0.873	0.869	0.909	0.896	0.920	0.898
mahalanobis	0.616	0.777	0.723	0.727	0.672	0.690	0.707

**Table 2:** Table showing the dependence of the results of sensitivity from the types of classification and the analyzed areas

	**LO**	**RO**	**LRO**	**LD**	**RD**	**LRD**	**LROD**
linear	0.688	0.739	0.711	0.757	0.718	0.714	0.715
diaglinear	0.701	0.712	0.684	0.757	0.704	0.742	0.701
quadric	0.623	0.739	0.644	0.771	0.619	0.707	0.652
diagquadric	0.636	0.657	0.644	0.771	0.619	0.678	0.663
mahalanobis	0.831	0.808	0.825	0.885	0.887	0.878	0.836

**Figure 4:**
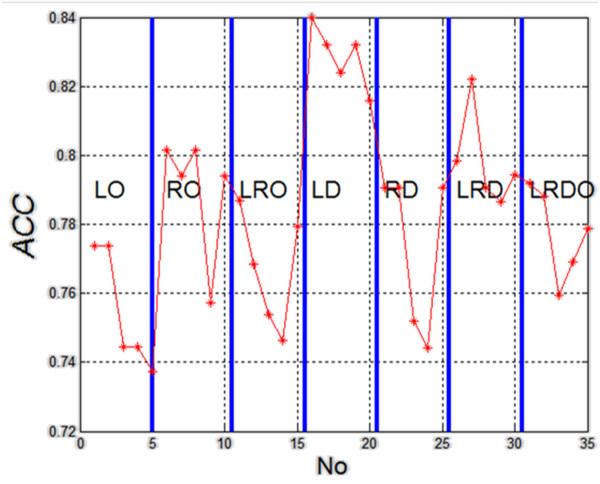
**The graph of accuracy (*ACC*) for subsequent analyzed cases (5 different types of classification and seven different configurations of the analyzed areas).** The graph is divided into methods of measurement. For each measurement method, classification was carried out in succession with five methods: linear, diaglinear, quadric, diagquadric, mahalanobis. The graph shows clearly that the type of the ROI, and not the type of classification, significantly affects the results. In this case, these are the images of the right longitudinal section of the thyroid (LD).

The results of specificity and sensitivity shown in Table 1 and Table 2 clearly indicate the linear method of classification of the left lobe in longitudinal section (LD). It can be also supported by making the calculation of accuracy (*ACC*= (*TP* + *TN*) / (*TP* + *TN* + *FP* + *FN*) ) for 35 cases (5 different types of classification and seven different configurations of the analyzed areas). The results are shown in Figure 4.

**Figure 5:**
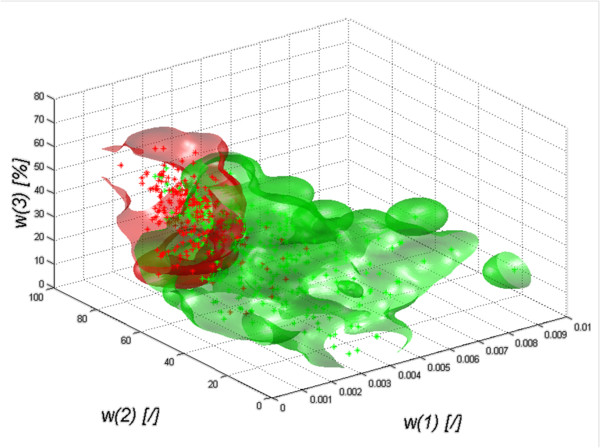
**The graph of classification objects (quadratic) of healthy subjects (red) from patients (green) depending on the features *w*(1), *w*(2) and *w*(3).** The graph shows a visual distinction (classification) between healthy subjects and patients. The graph also shows a common area which is included in both data groups (those of patients and healthy subjects). The presented graph is one of the possibilities to create a closed area covering the cases of healthy subjects and patients in the axes of the three features *w*(1), *w*(2) and *w*(3).

The presented results (Figure 4) unambiguously confirm the greatest diagnostic usefulness of ROI analysis of the left lobe in longitudinal section. The graph of classification objects (mahalanobis) depending on the features *w*(1), *w*(2) and *w*(3) is shown in Figure 5. Therefore, considerations of the impact of the method of marking the ROI, which will be presented later in the article, become interesting. ROI shifting and resizing indicated by an expert can influence greatly not only the features *w*(1), *w*(2) and *w*(3) but also specificity (*SPC*) and sensitivity (*TPR*), which will be presented in the following sections.

## Sensitivity to the change of parameters

The measured area (ROI), image *Ls*, underwent affine transformations in order to determine the dependence between the analyzed features *w*(1), *w*(2), *w*(3) and the size of the analyzed area as well as its position and rotation. The sensitivity analysis of these changes will be considered in subsequent sections.

This analysis was considered in two aspects:

- sensitivity of features *w*(1), *w*(2) and *w*(3) to affine transformations of the ROI,

- sensitivity of classification results to affine transformations of the ROI.

The analysis of changes in the value of *w*(1), *w*(2) and *w*(3) is important in this case because it points to their direct link with affine transformations (rotation, resizing and repositioning of the ROI). A direct comparison enables to assess the correctness of the formulation of features and their sensitivity to, for example, image rotation. This, in turn, enables to indicate which feature (and to what extent) depends on the position of the ultrasound head. It is also a condition to modify the formulation of a given feature so that it is only slightly dependent on the rotation.

Regardless of these results, the quality of the classification results for affine transformations -derived on the basis of all the features *w*(1), *w*(2) and *w*(3) – was assessed. The results demonstrate sensitivity of the algorithm which is considered as a measurement (diagnostic) method.

**Figure 6:**
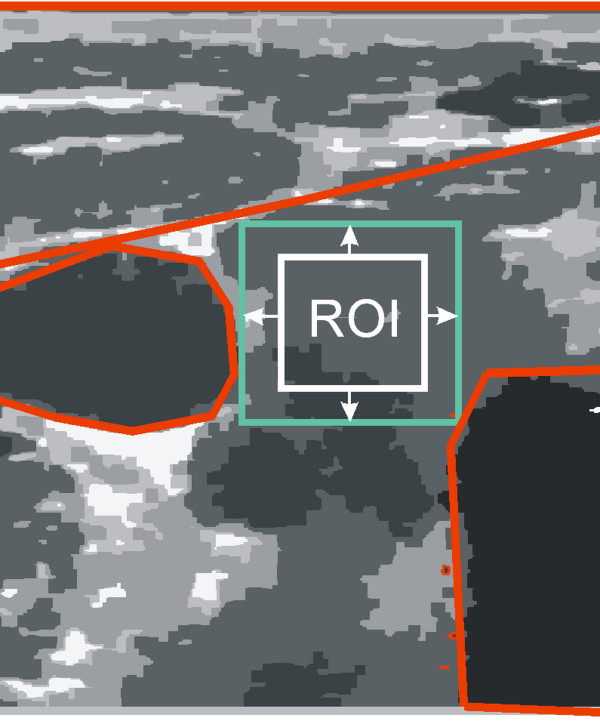
**The schematic diagram of the thyroid ultrasound image showing a typical distribution of the ROI (white) marked by a specialist physician and the distribution of adjacent organs (red).** Acceptable ranges of variation in the ROI shift, size and rotation are highlighted in green. On this basis, and analyzing all the images, restrictions on ranges of variation in the ROI position and size were specified.

Sensitivity assessment of the algorithm will be carried out for the changes in the position, size and rotation of the ROI. A range of changes in these parameters is limited by (Figure 6):

- organs immediately adjacent to the thyroid lobe,

- image borders - moved or enlarged ROI may not exceed the limits of the image,

- ROI cannot be smaller than 10×10 pixels - this limitation is recognized in the definition of the coefficients w(1), w(2) and w(3).

Therefore, rounded values of the changes in the ROI position in the range of ±20 pixels of the ROI and its size of 10×10 to 90×90 were adopted. These values do not result in a breach of any of the above restrictions on the ROI for any of the analyzed images.

The only correct position and size of the ROI are determined by a specialist physician. Results and their impact on the value of accuracy will be observed (calculated) during ROI shifting, resizing or rotating.

### The algorithm sensitivity to the resize of the marked area

**Figure 7:**
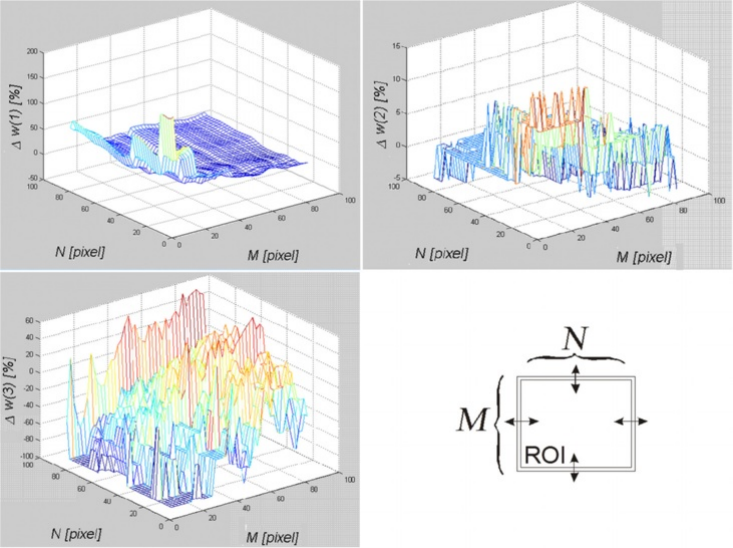
**Assessment of the algorithm sensitivity, *w*(1), *w*(2) and *w*(3) to the change of the number of rows and columns of the analyzed are in the range from *M*×*N*=10×10 to 90×90.** The graphs show the changes in individual features in response to changes in the size of the selected ROI. The graphs indicate that the features *w*(1) and *w*(2) are very sensitive to the size of the ROI. The smaller the ROI is, the bigger the measurement error of the values *w*(1) and *w*(2) gets. The feature *w*(3) is less dependent on the size of the ROI.

The measurement of the algorithm sensitivity to the change of size, resolution of the image *Ls* and, thus, the selected area was carried out on a healthy subject’s left thyroid lobe in transverse section (LD). For all the analyzed cases, the size of *Ls* images ranged from 24 to 81 rows and 20 to 92 columns. On this basis and considering specificity of obtaining the feature *w*(3) (the number of instances of the areas of size 8×8), the range of variation of the ROI was set. The selected area *Ls* was modified by changing its size from *M*×*N*=10×10 pixels to 90×90 pixels whereas the size of a properly selected area was 50×50 pixels. The change of size concerned independently modification of the number of rows and columns by 1 pixel. The minimum value of the ROI (10×10) resulted from the limitations of the algorithm operation in the case of the feature *w*(3). The maximum value (90×90) was limited by the edge of the image for the utmost part of the area. A USG specialist marked the area of 50×50 pixels which is the base size. For each modified area, the percentage changes (appropriate error as a percentage) of the features *w*(1), *w*(2), *w*(3) were measured. The graphs (Figure 7) show the obtained results.

The graphs (Figure 7) indicate that the feature *w*(3) is most sensitive to the change in the size of *Ls*. A small change in the number of rows and columns of the covered range indicated by the doctor influences considerably the value of the feature *w*(3). These changes concern tens of percent for the decrease or increase of the area by a few pixels. Changes to the feature *w*(1) are much milder and amount to several percent. When the area *Ls* increases, the value of the feature *w*(1) slightly changes. Only a significant reduction in the area *Ls* increases the error for the measurement of the feature *w*(1) up to 100%. Sensitivity of the feature *w*(3) to a change of the size of the area *Ls* looks completely different. The value of the feature varies by only a few percent in the full measured range. Discrete changes, visible on the graph, are due to the definition of the feature *w*(3) which is based on a histogram. Changes in the value of *w*(3) result from a change in the shape of the histogram. Therefore, the number of pixels of a given brightness and its proportion in relation to different brightness have to change. For the image *Ls*, it means that the texture changes. For this reason, changes in the size of the area *Ls* only slightly affect changes in the value of *w*(3).

### The algorithm sensitivity to the change of the marked area position

**Figure 8:**
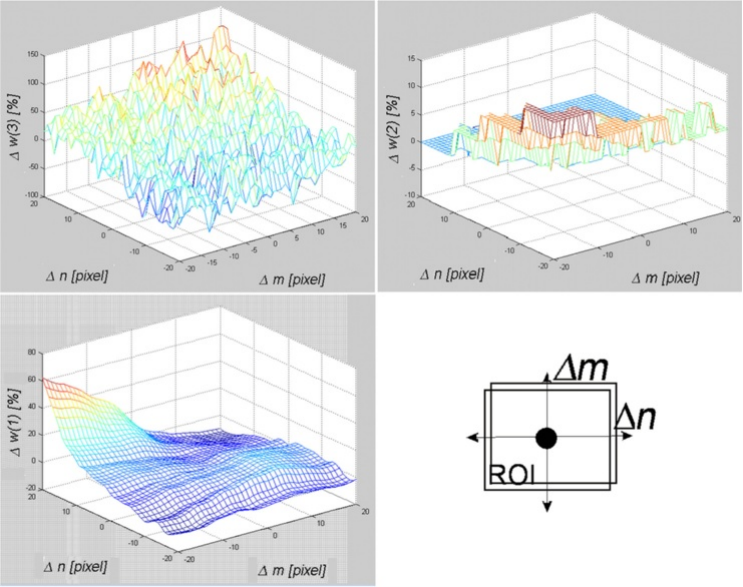
**Assessment of the algorithm sensitivity, *w*(1), *w*(2) and *w*(3) to the change of the analyzed area position in the range of ±20 pixels in rows (*m*) and columns (*n*).** The graphs indicate that the shift of the ROI in the range of −20 pixels in both axes increases the value of the feature *w*(2) by about 10%. For the same shift, the values of the feature *w*(1) and *w*(3) do not change. The feature *w*(3) increases its error to the value of 60% but only for the shift of -20 pixels in one axis and +20 pixels in the other.

The measurement of the algorithm sensitivity to the change of the *Ls* position in the thyroid ultrasound image was carried out by pushing the area marked by the expert in the range of ±20 pixels in the axes of rows (*m*) and columns (*n*). The range of ±20 pixels resulted from the variability in the content of the image for which the shift of more than 20 pixels resulted in an analysis of a neighboring organ. The ultrasound image was from a healthy subject and it was a transverse section of the thyroid left lobe. The results for the measured features *w*(1), *w*(2) and *w*(3) are shown in Figure 8.

It can be observed that values of the features *w*(1), *w*(2) and *w*(3) behave differently when the position of the *Ls* in the axes of rows and columns is changed. For the extreme positions, i.e. Δ*m*=20, Δ*n*=20 of the feature *w*(1), Δ*m*=−20, Δ*n*=−20 of the feature *w*(2) and Δ*m*=−20, Δ*n*=20 of the feature *w*(3), maximum values are achievable. Thus, a significant shift (more than 20 pixels) of the area *Ls* affects the results to a considerable extent. Globally, the feature *w*(3) is least sensitive to shifts of the area *Ls*.

### The algorithm sensitivity to rotation around its own axis

**Figure 9:**
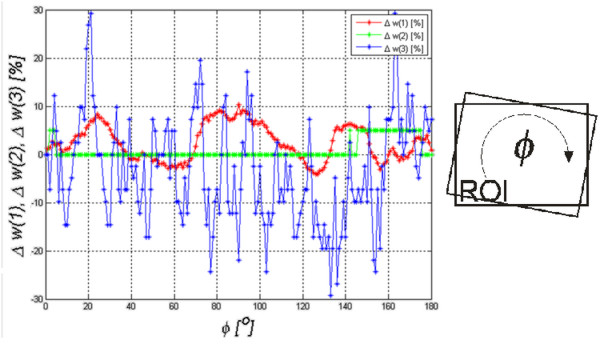
**Assessment of the algorithm sensitivity, *w*(1), *w*(2) and *w*(3), to the rotation of the analyzed area in the angular range ofϕ=0 to 180**^**o**^**by every 1**^**o**^**.** The rotation of the ROI affects the features *w*(1) and *w*(3) to the greatest extent. The change in the value of the error of the feature *w*(1) is cyclic and its frequency of changes is several times larger than the rotation.

Sensitivity to rotation of the analyzed area *Ls* is the last measured sensitivity to the change of parameters (of the features *w*(1), *w*(2) and *w*(3)). The analyzed area was rotated around an axis situated in the center of the area *Ls* in the angular range *ϕ*=0 to 180^o^ by every 1^o^ using a bilinear interpolation method. As in the previous measurements, the analyzed area concerned the thyroid texture in a healthy patient’s ultrasound image in the left transverse section. The results are shown in Figure 9.

The graph in Figure 9 shows that sensitivity to the rotation of the analyzed area is the highest for the feature *w*(3). The value of the feature *w*(2) changes slightly whereas the value of the feature *w*(1) changes oscillating. These oscillations result from the modification (due to rotation) of the *Ls* image content into new areas which contribute significantly to the value of STD and, therefore, to the value of the feature *w*(1).

In summary, the presented algorithm is least sensitive to the rotation of the area *Ls* and the feature *w*(2) is least sensitive to affine transformations (rotation, repositioning and resizing).

### Assessment of the classification method sensitivity to affine transformations of the ROI

Assessment of sensitivity presented in the previous sections is determined on the basis of the results obtained from the individual features *w*(1), *w*(2) and *w*(3). These results are meaningful when the features are considered separately. However, in the case of the presented algorithm for classification, they form a coherent whole equally influencing the decision function. Therefore, it becomes legitimate to analyze sensitivity of the classification method to the presented affine transformations – ROI shifting and resizing (*Ls*). *Ls* image rotation will not be analyzed because, as it has been proved in previous sections, its influence on the results is negligibly small.

**Figure 10:**
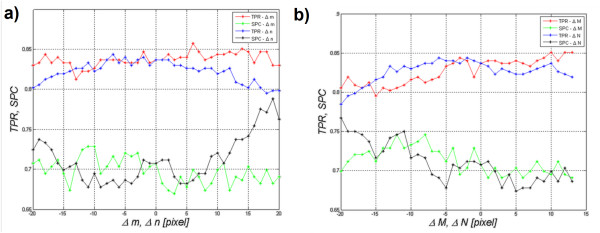
**Assessment of the algorithm sensitivity (specificity (*SPC*), sensitivity (*TPR*)) to changes in: a) ROI position in the axes of rows and columns (Δ*m*, Δ*n*) in the range of ±20 pixels and b) ROI size in the axes of rows and columns (Δ*M*, Δ*N*) in the range of ±20 pixels.** The presented graph **a**) suggests many conclusions concerning the impact of the change in the position of the ROI on sensitivity and specificity. For example, the ROI shift in the range of ±10 pixels in the row or column axis slightly affects the values of specificity and sensitivity (changes at below 0.05). The figure **b**) shows that an increase in the size of the ROI by 13 pixels in rows or by 7, 8 pixels in rows and columns causes a significant increase in specificity and sensitivity by approximately 0.03. The analysis of this graph **a**) and the graph **b**) indicates that the areas were properly marked by an expert. In another case, the maximum values of sensitivity and specificity for the shift 0 and the change of size 0 of the marked ROI were not visible.

The figure below (Figure 10a)) shows the impact of a shift in the area *Ls* in the range of ±20 pixels in the axes of rows or columns on the values of *SPC* and *TPR* (Δ*m*=±20 or Δ*n*=±20). Figure 10b), on the other hand, shows the impact of changes in the size of the ROI on the obtained results (*SPC* and *TPR*). Since the ROI sizes are different for different ultrasound images, values of the changes *M*×*N* are given as differences with respect to the original size in the range of ±20 pixels, i.e.: Δ*m*=±20 or Δ*n*=±20. Reducing the area did not result, in any case, in the ROI smaller than 5×5 pixels. Moreover, at a magnification, the ROI did not exceed the limits of an ultrasound image.

**Figure 11:**
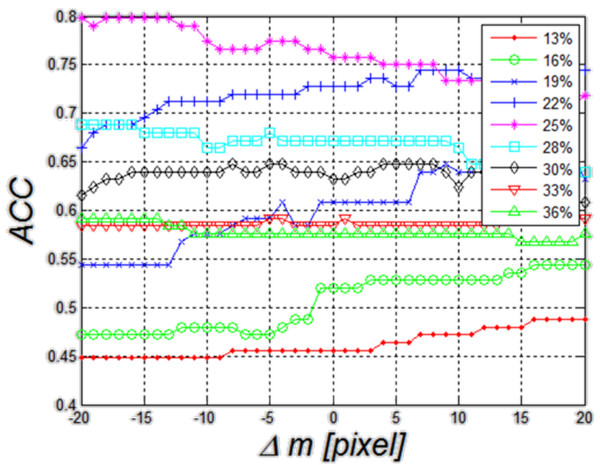
**Graph of changes in the*ACC*value as a function of Δ*m*for five different threshold settings for the first compared method (method 1).** These results are related to the impact of ROI repositioning on the value of *ACC*. Threshold values are mean values of echogenicity in the ROI. The best results were obtained with a threshold of 25%. For this value, changes in *ACC* are less than 10%.

**Figure 12:**
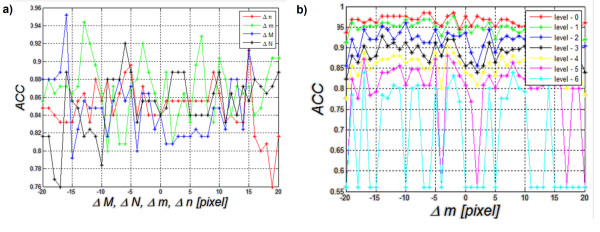
**Graph of changes in*ACC*as a function of Δ*m*, Δ*n*, Δ*M*and Δ*N*when decision trees are used as a classifier a) and graph of changes in*ACC*as a function of Δ*m*for each level of the decision tree cutting b)- this is the second compared method (method 2).** Changes (graph a) in the position and size of the ROI affect to an increasing extent the dynamics of changes in *ACC*. For the basic setting Δ*m*=Δ*n*=Δ*M*=Δ*N*=0, *ACC* is equal to 84%. The further from the basic setting 0 pixels, the higher the dynamics of changes in *ACC* becomes. The value of the level 0 (graph b) means no tree pruning. Trees are pruned based on an optimal pruning scheme that first pruned branches give less improvement in error cost. For most of the created decision trees, changes in *ACC* are in the range of 5%. When decision trees are cut too much, they lose their ability of correct classification. Such a situation is visible in the chart below (level 6) where *ACC* changes from 85% to 55%.

The graph in Figure 10a) shows that changes in ROI position in the range of ±20 pixels affect specificity and sensitivity to a lesser extent. When analyzing both Figure 10a) and b), some interesting properties and characteristics of the measurements can be noticed:

-ROI shift in the range of ±10 pixels in the row or column axis slightly affects the results of specificity and sensitivity (changes of less than 0.05),

-for a shift to the left or to the top by 10 pixels, *SPC* increases by approximately 0.03,

-an increase in the size of the ROI by 13 pixels in rows or by 7 to 8 pixels in rows and columns causes a significant increase in specificity and sensitivity by approximately 0.03.

In conclusion, the choice of the area conducted by the expert and the algorithm are very good. ROI shifts in the range of ±10 pixels in the row or column axis as well as a decrease or increase in the ROI do not significantly affect the results. Therefore, the algorithm is resistant to fluctuations of the ROI (of both position and size) and its rotation (as demonstrated in the previous section).

## Comparison with other results

In the literature described in the introduction [1-39], authors present several original methods of ultrasound image analysis. These methods are very interesting from the point of view of an ultrasound operator as they increase the accuracy and efficiency of diagnosis. Verification of sensitivity of the presented algorithms to changes in parametres, such as position, size and rotation of the ROI, is also an important feature for operators. This sensitivity analysis is important from the point of view of medical practice and interindividual variation. These elements may significantly influence the obtained results which testify to the quality of the algorithm. It may be that the advantage of one approach over the other forces highly accurate and precise indication of the ROI.

Comparing the described algorithm with other algorithms, a few common features may be found:

-the histogram analysis of our algorithm fulfills a similar function as a classical analysis of the histogram described in paper [10]. However, in that paper only one feature is taken into account, namely *w*(2) which is the minimum brightness, but after the removal of noise. Noise is defined as pixels whose sum is less than 20% of the calculated maximum amount of pixels.

-the analysis of the features of our algorithm is similar to the analysis of another set of features (entropy, sum variance and mean value) presented in paper [22]. Accuracy obtained there reaches 93.6%. However, the example given does not apply to Hashimoto’s disease.

-comparison of methods of Co-occurrence matrix with the Radon transform and Muzzolini’s spatial features is shown in paper [19]. However, the results shown do not relate directly to Hashimoto’s disease and do not analyze the impact of changes in the position of ROI on the obtained results.

-simple analysis of the areas associated with Hashimoto’s disease is shown in paper [42]. The results were obtained depending on the analysis method; sensitivity in the range of 71% to 88% and specificity in the range of 67% to 91%. These results are comparable with the results obtained with our algorithm, i.e. sensitivity 76% and specificity 95%. It should be noted that in the quoted paper [42], ROI areas were carefully selected by experts and some of the artifacts were manually eliminated.

In addition, the results of sensitivity, specificity and accuracy obtained from this discriminant analysis were compared in detail with other known methods [10,12,39]. Calculations were performed for the same group of 73 patients with Hashimoto’s disease and 59 healthy subjects. The images concerned only the left thyroid lobe in cross section (LD).

The following results were obtained:

Method 1: a classification method based on thresholding of mean values of brightness levels [10]– sensitivity 92% and specificity 40%. *ACC*=76%,

Method 2: a method that uses decision trees described in paper [39] - sensitivity 88% and specificity 76% *ACC*=84% for a pruned decision tree,

Method 3: a discriminatory classification method proposed in this paper - sensitivity 76% and specificity 95%. *ACC*=84%.

The exact differences between the three methods are described in detail below.

**Figure 13:**
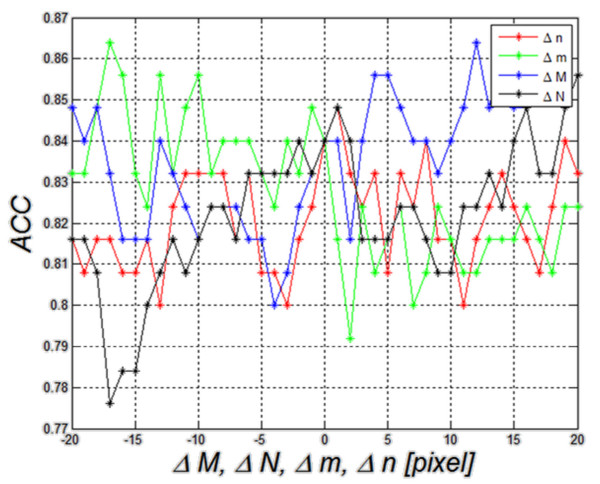
**Graph of changes in *ACC *as a function of Δ*m*, Δ*n*, Δ*M *and Δ*N *for linear dyscryminat analisys– this is the third described method (method 3).** The graph shows that the value of *ACC* is 84% for Δ*m*=Δ*n*=Δ*M*=Δ*N*=0. The other values of *ACC* change in the range of about 5% for ROI shifts and size changes. The direction of these changes is different and it is difficult to clearly link it to ROI shift and resize.

Method 3. The last results of changes in accuracy as a function of Δ*m* are shown in Figure 13 and they concern the third method described in this paper. Changes Δ*m*, Δ*n*, Δ*M*, Δ*N* within the same limits (±20 pixels) affect the result of accuracy by approximately ±5%. The resulting range of accuracy variation (5%) is the smallest of all the compared methods.

**Figure 14:**
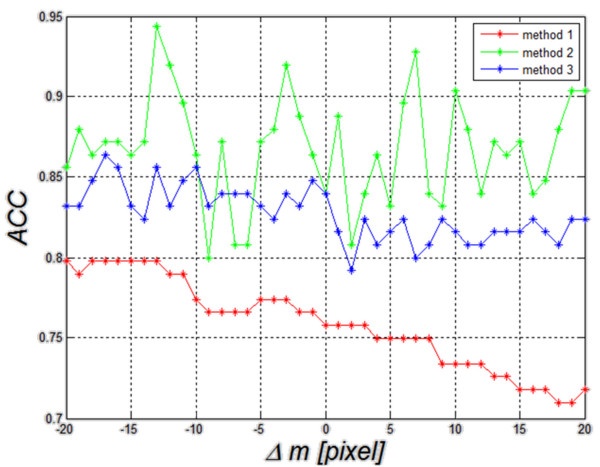
**Graph of changes in *ACC *as a function of Δ*m *for the three compared methods: method 1- thresholding, method 2- decision trees and method 3- discriminant analysis.** The worst results of classification of patients with Hashimoto’s disease (*ACC*=77%) were obtained for the method of thresholding of echogenicity average levels (red). Comparable results for Δ*m*=0 were obtained for decision trees and discriminant analysis. Here discriminant analysis is characterized by smaller changes in accuracy for different positions of the ROI.

In order to better compare the three methods (method 1, 2 and 3), they are shown jointly in Figure 14. The worst results of *ACC* (*ACC*=76%) are obtained for the method of thresholding (method 1) of echogenicity average levels (Figure 14). Changes in *ACC* for changes Δ*m*=±20 pixels range from 71% to 80%. When decision trees are used as a classifier (method 2), *ACC* variation range comprised between 80% and 95%. It was the best possible result obtained for the changes Δ*m*. For Δ*m*=0, it was 84%. Much smaller changes in *ACC* for fluctuations of Δ*m* can be observed in the discriminant method (method 3). For this method, changes in *ACC* are much smaller in the full range of Δ*m* variation and they range from 79% to 87% of *ACC*. For Δ*m*=0, *ACC* is 84%. Discriminant analysis is characterized by minor changes in accuracy for different positions of the ROI as compared to the method 2 that uses decision trees.

Therefore, in the assessment of Hashimoto’s disease, more than one feature needs to be taken into account. Moreover, DICOM files should be analyzed directly and one of the two of the compared classifiers should be used (discriminant analysis or decision trees- method 3 and 2). Not only the absolute values of *ACC* but also the dynamics of their changes for small ROI displacements should be taken into account when analyzing the changes in results caused by ROI displacements.

Method 1. The first method is based on thresholding of echogenicity mean value (described in detail in [10] ). When applied to these data, it enables to obtain a result of ACC equal to 76% for the gray level threshold set to 25% of luminance (Figure 11). The range of average gray levels in the ROI for the analyzed cases was between 10% and 39% of saturation. Therefore, the graph shown in Figure 11 was carried out for different values of the threshold changed in the range of 13% to 36% in increments of 2.9% (assuming a step which is the tenth part of the range of 39%-10%). It can be observed that for the threshold value of 25%, shift of the ROI in the range of ±20 pixels affects significantly the value of accuracy– *ACC* changes by 7%. For the other threshold settings, the value of changes of Δ*m* remains at a similar level, not exceeding 10%. In no sequence, a maximum for the value of Δ*m*=0 is visible. Changes in the accuracy for different Δ*m* do not have a well-defined direction of growth. Thus, it can be ultimately assumed that in the method of thresholding of echogenicity average level, ROI repositioning affects the result of accuracy to the extent of less than 10%.

Method 2: Another method uses decision trees (described in detail in [39] ). When applied to the collected data, it enables to obtain accuracy at 84%. In this case, accuracy variation was evaluated as a function of changes in ROI size and shift (Δ*m*, Δ*n*, Δ*M*, Δ*N*). The results for the pruned decision tree are shown in Figure 12a). The best tree is the one that has a residual variance that is no more than one standard error above the minimum value along the cross-validation line. Figure 12a) shows that changes in accuracy for changes in the values Δ*m*, Δ*n*, Δ*M* and Δ*N* are similar to the ones observed for echogenicity thresholding method and change by about 10%. The results also show a range of changes in the value of accuracy for each shift or resize of the ROI. The greater changes in the size or shift of the ROI are, the higher accuracy rate of change becomes. For example, for Δ*M* revised from the value of −16 to −15 pixels, the change in *ACC* reaches 16% (95%-79%). For Δ*M* as well as Δ*m*, Δ*n* and Δ*N* close to zero, *ACC* changes are smaller and reach the values of 5, 10%.

Narrowing the analysis to observation of accuracy changes only as a function of Δ*m*, the impact of pruning the decision tree on the results is shown in Figure 12b). The degree of cutting the decision tree is dependent on the level ranging from 0 to 6 where level = 0 means no tree pruning. Trees are pruned based on an optimal pruning scheme that first pruned branches give less improvement in error cost. It can be seen that accuracy values vary depending on the degree of cutting the decision tree. Δ*m* changes affect the value of accuracy by 5% for the first level values. When the decision tree is pruned too much, it loses its ability of classification and the error of accuracy reaches 30%.

## Summary

This paper presents the influence of a measurement method of echogenicity in the diagnosis of Hashimoto’s disease, with a particular reference to the assessment of the algorithm sensitivity to a change in the ROI position. Classification was performed using a discriminant analysis for the following five options: linear, diaglinear, quadratic, diagquadratic and mahalanobis. Transverse and longitudinal sections of the thyroid right and left sides were analyzed. The analysis showed that the highest accuracy was obtained for the longitudinal section (LD) with the linear method, obtaining sensitivity = 76%, specificity = 95% and *ACC* = 84%. The impact of changes in the location of the ROI on the results was shown in one example and, separately, for all the analyzed cases. A change in the ROI position has the greatest impact on the value of features *w*(1) and *w*(3). The feature *w*(3) showed the greatest dependence on both the ROI position and also change of its size in the measured range of ±20 pixels. The percentage changes in the feature *w*(3) in the measured range Δ*m*=±20 pixels and Δ*n* =±20 pixels exceed 100%, while the changes of the feature *w*(2) amount to 5, 10%. The change in the value of *w*(1) is between 50% and 60%. The analysis of the results (mainly in Figure 9), confirms low dependence (below 30%) of any feature *w*(1), *w*(2) or *w*(3) on the ROI rotation in the range of 0 to 180^o^. A significant variation in the features *w*(3), *w*(2) or *w*(1) is not meaningful in relation to changes in sensitivity and specificity for the analyzed group of patients. Sensitivity assessment studies confirm that changes in the ROI position and size have little effect on sensitivity and specificity. *SPC* changes from 60% to 74% and *TPR* from 75% to 83% in the analysis of all cases of RLOD. Comparing the obtained results with other methods (method 1,2) known from the literature is also interesting. In the case of the classification method which uses decision trees [39] - method 2, the dynamics of *ACC* changes was at 15% (from 80% to 95%) for the full ROI displacement by Δ*m*=±20. In the case of the method of thresholding (method 1) of echogenicity average levels, *ACC* was 76% for Δ*m*=0 and the variation range of *ACC* was from 71% to 80% for Δ*m*=±20 pixels.

## Competing interests

The authors declare that they have no competing interests.

## Authors’ contributions

RK and AK suggested the algorithm for analysing and processing images, implemented it and analysed the ultrasound images. JM conducted the study, participated in the collection of literature and supervised the base at the time of collecting material. AW also conducted the study and prepared material for analysis. WZ came up with the concept of image analysis in Hashimoto’s disease, collected and analyzed literature, conducted studies, correlated the images with the results and participated in the analysis. ZW and WW coordinated the work of the whole team and consulted all the stages of the project. All authors have read and approved the final manuscript.
